# Lethal Legionella Pneumonia With Bacteremia Due to Serotype 3 Co-infected With Staphylococcus aureus and Haemophilus influenzae

**DOI:** 10.7759/cureus.95860

**Published:** 2025-10-31

**Authors:** Yuzo Obata, Masafumi Seki, Shuhei Miyata, HIkari Omi, Hiroshi Ogawa, Yoshitaka Ooya, Ayumu Masuoka, Futoshi Kotajima, Kotaro Mitsutake, Yoshitaka Inoue, Manabu Nemoto, Masahito Kaji

**Affiliations:** 1 Department of Trauma and Emergency Acute Medicine, Saitama Medical University International Medical Center, Hidaka, JPN; 2 Division of Infectious Disease and Infection Control, Saitama Medical University International Medical Center, Hidaka, JPN; 3 Emergency and Acute Medicine, Saitama Medical University International Medical Center, Hidaka, JPN; 4 Respiratory Support Team and Department of Emergency Pediatrics, Saitama Medical University International Medical Center, Hidaka, JPN; 5 Respiratory Support Team and Department of Intensive Care Medicine, Saitama Medical University International Medical Center, Hidaka, JPN; 6 Department of Infectious Diseases and Infection Control, Saitama Medical University International Medical Center, Hidaka, JPN

**Keywords:** beta-lactamase negative haemophilus influenzae (blnar), legionella pneumophila, methicillin-susceptible staphylococcus aureus (mssa), serotype 3, venovenous extracorporeal membrane oxygenation

## Abstract

A 66-year-old man with no relevant medical history developed pneumonia. Urine antigen tests for pneumococcus and *Legionella* serotype 1 (the most common serotype) were negative, but sputum culture was positive for methicillin-susceptible *Staphylococcus aureus* (MSSA) and β-lactamase-negative *Haemophilus influenzae *(BLNAR). The patient was treated with meropenem and azithromycin, but an aerobic blood culture became positive on day 4, and mass spectrometry analysis indicated the presence of *Legionella* species. Subculture on buNrNL charcoal yeast extract (BCYE)-α agar identified *Legionella pneumophila* serotype 3. The patient was diagnosed with pneumonia caused by MSSA and BLNAR, as well as pneumonia/bacteremia due to *Legionella* *pneumophila* serotype 3. Despite changing the antibiotics to meropenem plus lascufloxacin and adding hydrocortisone and venovenous extracorporeal membrane oxygenation (V-V ECMO), the patient died. In patients with severe pneumonia, co-infection with *Legionella* species, including rare serotypes, should be considered, and universal rapid antigen tests are necessary to ensure rapid diagnosis and appropriate treatment.

## Introduction

*Legionella* species account for approximately 2-15% of community-acquired pneumonia (CAP) cases [1,2]. *Legionella* pneumonia can lead to acute respiratory distress syndrome (ARDS) [3] and severe respiratory failure with a reported mortality of up to 32.4% in patients with *Legionella* infection who require intensive care [4,5]. Bacteremia due to *Legionella* species is rare, occurring most commonly in immunocompromised hosts such as those with hematological disease/transplantation [6,7], and can become severe [8].

The 48 species in the genus *Legionella* comprise 70 distinct serogroups; however, *L. pneumophila* serogroup 1 might be almost 80% of all urine antigen-confirmed and/or culture-confirmed cases [1]. Universal antigens that could diagnose all *L. pneumophila *had not been discovered. Therefore, *Legionella* cases by the less common strains are reported infrequently because they are rare, and diagnostic reagents have been lacking [3]. In fact, delayed diagnosis of several *Legionella* pneumonia cases due to the *L. pneumophila* other than serotype 1 had been reported and became severe because of delayed treatment [5,7]. Furthermore, from the beginning, many clinicians did not notice the existence of these kinds of *L. pneumophila* other than serotype 1. These few understandings and gaps in the distribution of these strains might also contribute to the increase in mortality [3].

This report discusses the case of an adult patient with no general comorbidities who presented with lobar pneumonia that initially appeared to be due to infection with methicillin-susceptible *Staphylococcus aureus *(MSSA) and β-lactamase-negative and ampicillin-resistant (BLNAR) *Haemophilus influenzae*. Meropenem plus azithromycin treatment was ineffective. A rapid urine antigen test for* L. pneumophila* serotype 1 was negative, but *L. pneumophila *serotype 3 was isolated from the blood culture.

## Case presentation

**Figure 1:**
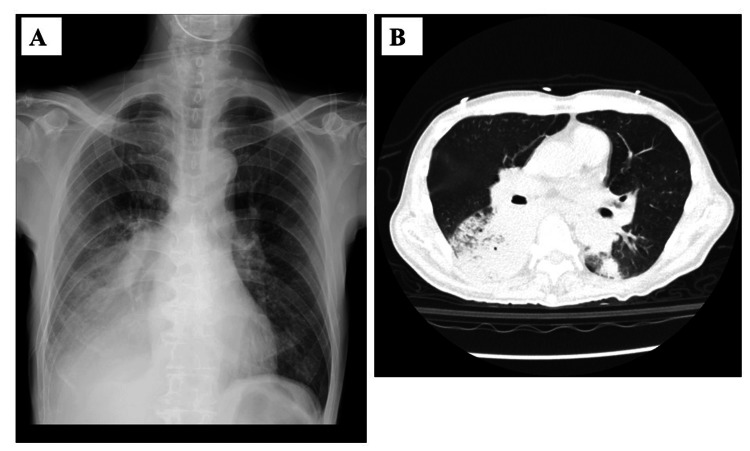
Chest X-ray (A) and computed tomography (B) images of the patient with severe pneumonia and bacteremia due to Legionella pneumophila serotype 3 and co-infection with methicillin-susceptible Staphylococcus aureus (MSSA) and b-lactamase-negative Haemophilus influenzae (BLNAR). Massive infiltration shadows are seen mainly in the right lung and left lower lung.

A 66-year-old man with no past medical history developed general malaise, loss of consciousness, and dyspnea in February 2024. He had no history of underlying diseases, including diabetes mellitus, but mentioned visiting hot springs or circulating baths, which are sources of *Legionella* infections. The patient’s temperature was high (38.3 °C), and consciousness was not clear (Glasgow Coma Scale: E2V3M4) on day 0. On presentation (day 0), chest X-ray and computed tomography (CT) showed lobar infiltration shadows with slight ground-glass opacities (GGOs) in the right lung field and nodular shadows in both lung fields (Figure 1A, 1B). Nasal swabs were negative by the polymerase chain reaction (PCR) for SARS-CoV-2 (Cobas SARS-CoV-2; Roche, Basel, Switzerland) test and for influenza using the influenza rapid antigen test (immunochromatography) (QuickNavi; Otsuka Co. Ltd., Tokyo, Japan).

Laboratory data on admission to our hospital (day 0) are shown in Table 1. Arterial oxygen saturation (SpO_2_) was 92% (O_2_ mask, 15 L), and intubation ventilator management was initiated in the intensive care unit (ICU). The patient presented with lobar pneumonia and severe hypoxia, renal failure, and high inflammatory status. Invasive pneumococcal pneumonia and/or *Legionella* pneumonia were suspected; however, rapid urine antigen tests for *Streptococcus pneumoniae* and *L. pneumophila* serotype 1 (BinaxNOW, Abbott, Abbott Park, IL) were both negative. He has no past history of bacterial isolation from his sputum.

**Table 1:** Laboratory data of the patient

Items	Data	Normal range
White blood cells (WBC)	3.38 × 10^3^/μL	3.3-8.6
Neutrophils	94%	42.4-75
Lymphocytes	2.40%	18.2-47.7
Monocytes	1.40%	3.3-9
Eosinophils	0.30%	0.4-8.6
Basophils	1.20%	0.2-1.4
Hemoglobin	13.4 g/dL	13.7-16.8
Platelet	26.9 × 10^ 3^/μL	15.8-34.8
Serum creatinine	1.83 mg/dL	0.65-1.87
Blood urea nitrogen	49.9 mg/dL	8-20
Aspartate aminotransferase (AST)	50 U/L	13-39
Alanine aminotransferase (ALT)	33 U/L	10-42
C-reactive protein (CRP)	28.9 mg/dL	0-0.14
Procalcitonin (PCT)	68 ng/mL	<0.05

Meropenem 1 g x 3/day and azithromycin 0.5 g x 1/day with hydrocortisone (200 mg/day) were administered intravenously, according to our hospital’s treatment protocol for patients admitted with severe pneumonia. Two days later, sputum examination identified MSSA and BLNAR. Furthermore, anaerobic blood culture became positive on day 4, and mass spectrometry analysis revealed the presence of *Legionella* species. Therefore, severe pneumonia due to MSSA and BLNAR was suspected, in addition to *Legionella* pneumonia/bacteremia, and azithromycin was changed to lascufloxacin (LSFX) 300 mg x 1/day on the first day, followed by 150 mg x 1/day intravenously to cover the three pathogens, such as MSSA, BLNAR, and *Legionella* powerfully, despite the negative rapid urine antigen test for *Legionella* at admission. Seven days later, a subculture of bacteria isolated from the patient’s admission blood culture on buNrNL charcoal yeast extract (BCYE)-α agar revealed *L. pneumophila* serotype 3. Despite administration of antibiotics, including LSFX to which MSSA, BLNAR, and *L. pneumophila* serotype 3 were all susceptible, in addition to venovenous extracorporeal membrane oxygenation (V-V ECMO) performed from day 7, the patient's condition, including chest X-ray findings, did not improve. The laboratory data at day 10 also showed a still high inflammation status, such as white blood cell count of 20.69 x10^3^/μL, C-reactive protein of 10.4mg/dL, and lactate dehydrogenase of 797 U/L, and we added rifampicin 450 mg by a nasogastric tube. However, the patient became shocked again on day 14 and died suddenly.

## Discussion

*Legionella* species are lethal pathogenic bacteria that induce severe pneumonia. Among *Legionella* species, *L. pneumophila *is the most pathogenic, with serotype 1 the most prevalent and considered a representative group as it is the cause of 60-80% of cases of lethal pneumonia [1]. Rapid antigen tests have been developed to detect serotype 1 alone [2].

In this report, we present a severe case of pneumonia with bacteremia caused by* L. pneumophila* serotype 3 in a patient co-infected with MSSA and BLNAR. Of these, *L. pneumophila* type 3 might have been the most dominant because this pathogenic bacterium was isolated from both sputum and blood, whereas MSSA and BLNAR were detected only in sputum. *L. pneumophila *serotype 3 is also pathogenic. A recent study reported the case of a 67-year-old woman with severe hypoxia due to diffuse right-sided pneumonia and reddish bronchoalveolar lavage fluid consistent with diffuse alveolar hemorrhage, in the absence of airway inflammation and purulence [5]. Gram staining showed scant white blood cells with no organisms, but *Legionella* PCR testing of bronchoalveolar lavage fluid was positive, and *Legionella* cultures were obtained. These findings suggest that *L. pneumophila *serotype 3 is pathogenic and can induce severe lung damage. Interestingly, that patient also showed a negative *Legionella* urine antigen test, although the authors also suspected *Legionella* and/or pneumococcal pneumonia due to extensive pulmonary infiltrates and elevated inflammatory markers on blood tests, similar to the present case. These data indicate that it is critical to obtain an accurate diagnosis and strong suspicion for* L. pneumophila *serotype 3 from the early phase in patients with severe pneumonia.

For many years, the BinaxNOW (Abbott, IL, USA) rapid urine antigen test has been in common use worldwide for *L. pneumophila* serotype 1 [9], but another rapid point-of-care test (POCT) is needed to detect *L. pneumophila* serotypes other than serotype 1 that are also possible pathogens of severe community-acquired pneumonia. Recently, not only the conventional *L. pneumophila *serogroup 1 lipopolysaccharide, but also all serogroups of *Legionella* species have been able to be detected by the Ribotest *Legionella* kit (Ribotest; Kyokuto Seiyaku Kogyo, Tokyo, Japan) using universal antibodies that targeted the *L. pneumophila* ribosomal protein L7/L12. The sensitivities of various kits using in vitro culture-soluble antigen extracts of ATCC strains and 22 clinical isolates of *Legionella* species were assessed in a previous study, and the authors found that *L. pneumophila* non-serogroup 1 and legionellae of other species could not be detected by the four conventional kits, including BinaxNOW (Abbott, IL, USA); however, the Ribotest *Legionella* kit detected them with a sensitivity of 10^5^-10^8^ CFU/mL [10]. These data suggest the high potential of Ribotest as a rapid diagnostic test for pneumonia caused by other *Legionella* species. Indeed, following our experience with the present lethal case caused by* L. pneumophila *serotype 3 infection, we have replaced BinaxNOW with the Ribotest as the POCT at the bedside and in the emergency department at our hospital.

In addition, the present patient had co-infection with MSSA and BLNAR as well as *L. pneumophila* serotype 3. Co-infection may accelerate the severity of pneumonia. In a previously reported case of bacteremia caused by both *Helicobacter cinaedi *and *L. pneumophila* serogroup 2 in a 74-year-old woman, the patient was intubated and transferred to the intensive care unit, where levofloxacin and tazobactam/piperacillin were administered, and the patient gradually recovered [7]. The combination of *L. pneumophila* serotype 2 and *H. cinaedi *might be milder than triple infection with *L. pneumophila* serotype 3, MSSA, and BLNAR, as in the present case. In our case, the patient might actually show very high inflammation marker status and severe respiratory failure, compared with either MSSA, BLNAR, or *L. pneumophila* alone.

In the present case, we administered a novel fluoroquinolone, LSFX, that has recently been used as a drip infusion for treating pneumonia due to anaerobes, and showed good penetration for intrapulmonary lesions [11]. Furthermore, successful treatment with LSFX has been reported in three hospitalized patients with *Legionella* pneumonia [12]. These results suggest that LSFX has similar efficacy and might even be superior to levofloxacin for the treatment of severe *Legionella* pneumonia. The present patient also received V-V ECMO to improve oxygenation, but did not improve and died due to severe lung damage and triple infection, including *Legionella* bacteremia.

## Conclusions

This report presents a severe case of pneumonia with bacteremia due to *L. pneumophila *serotype 3 and co-infection with MSSA and BLNAR. The conventional urine antigen test did not detect *Legionella* on admission, leading to delayed administration of appropriate antibiotics such as LSFX and of V-V ECMO support, and the patient did not survive. Therefore, it is necessary to implement universal rapid antigen tests for *L. pneumophila *serotypes other than serotype 1 and for rare *Legionella* species other than *L. pneumophila*. We need to use not only β-lactams but also fluoroquinolones to avoid treatment delay when we see severe pneumonia suggesting *Legionella* pneumonia.
